# Brain health is essential for smooth economic transitions: towards socio-economic sustainability, productivity and well-being

**DOI:** 10.1093/braincomms/fcae360

**Published:** 2024-10-30

**Authors:** Olivia Nail-Beatty, Agustin Ibanez, Rym Ayadi, Pawel Swieboda, Alfred K Njamnshi, Jo-An Occhipinti, William Hynes, Burcin Ikiz, Laura Castro-Aldrete, Kelly O’Brien, Michael L Platt, Shazia Adalat, Jafri Malin Abdullah, Rajinder K Dhamija, Zul Merali, Cyprian Mostert, Debbie Beck, Shekhar Saxena, Mohamed Salama, Omnia M Abdelraheem, Frederic Destrebecq, George M Slavich, Bello Shehu, Graham Fieggen, Paul M Ghogomu, Claudio L A Bassetti, Harris A Eyre

**Affiliations:** Neuro-Policy Program, Center for Health and Bioscience, Baker Institute for Public Policy, Rice University, Houston, TX 77006, USA; BrainLat, University Adolfo Ibanez, Santiago 7941169, Chile; Global Brain Health Institute (GBHI), Dublin D02 PN40, Ireland; Euro-Mediterranean Economists Association, Barcelona 08025, Spain; NeuroCentury, Brussels 1060, Belgium; Global Brain Health Institute (GBHI), Dublin D02 PN40, Ireland; Euro-Mediterranean Economists Association, Barcelona 08025, Spain; NeuroCentury, Brussels 1060, Belgium; International Centre for Future Generations, Brussels 1040, Belgium; FIPRA, Brussels 1040, Belgium; European Policy Centre, Brussels 1000, Belgium; Brain Research Africa Initiative (BRAIN), Yaoundé 25625, Cameroon; Neuroscience Lab, Faculty of Medicine and Biomedical Sciences, The University of Yaoundé, Cameroon; Department of Neurology, Central Hospital Yaoundé, Yaoundé, Cameroon; Brain Research Africa Initiative (BRAIN), Geneva, Switzerland; Mental Wealth Initiative, The University of Sydney, Sydney 2050, Australia; Institute for Global Prosperity, University College London, London W1T 7NE, UK; Santa Fe Institute, Santa Fe, NM 87501, USA; World Bank, Washington, DC 20433, USA; EcoNeuro, Los Angeles, CA, USA; Baszucki Group, San Mateo, CA 94401, USA; International Neuro Climate Working Group, Columbia University, New York, NY 10032, USA; Psychology Today, New York, NY 10010, USA; Department of Laboratory Medicine, Karolinska Institutet, Stockholm 141 52, Sweden; USAgainstAlzheimer’s, Washington, DC 20015-2604, USA; Department of Neuroscience, Wharton Neuroscience Initiative, University of Pennsylvania, Philadelphia, PA 19104, USA; Evelina London Children’s Hospital, Guy’s and St Thomas’ NHS Foundation Trust, London SE1 7EH, UK; Department of Neurosciences, Brain and Behavior Cluster, Universiti Sains Malaysia, School of Medical Sciences/Hospital Universiti Sains Malaysia, Kubang Kerian, Kelantan 16150, Malaysia; Academy of Science Malaysia, 20th Floor, Wisma Matrade, Kuala Lumpur 50480, Malaysia; Neurology, Institute of Human Behavior and Allied Sciences, New Delhi 110095, India; Mind and Brain Institute, The Aga Khan University, Karachi, Pakistan; Mind and Brain Institute, The Aga Khan University, Karachi, Pakistan; Perkins & Will, Dallas, TX 75201, USA; Chan School of Public Health, Harvard University, Boston, MA 02115, USA; Institute of Global Health and Human Ecology, The American University of Cairo, Cairo, Egypt; International Neuro Climate Working Group, Columbia University, New York, NY 10032, USA; European Brain Council, Brussels 1000, Belgium; Department of Psychiatry and Biobehavioral Sciences, University of California, Los Angeles, CA 90024, USA; Regional Centre for Neurosurgery, Usmanu Danfodiyo University Teaching Hospital, Sokoto, Nigeria; Department of Surgery and Neuroscience Institute, Faculty of Health Sciences, University of Cape Town, South Africa; Neuroscience Lab, Faculty of Medicine and Biomedical Sciences, The University of Yaoundé, Cameroon; Neurology Department, Inselspital (CLAB), University of Bern, Bern 3008, Switzerland; Swiss Brain Health Plan (CLAB), Bern 3008, Switzerland; Neuro-Policy Program, Center for Health and Bioscience, Baker Institute for Public Policy, Rice University, Houston, TX 77006, USA; Global Brain Health Institute (GBHI), Dublin D02 PN40, Ireland; Euro-Mediterranean Economists Association, Barcelona 08025, Spain; Global Brain Health Institute, University of California San Francisco (UCSF), San Francisco, CA 94158, USA; Department of Psychiatry and Behavioral Science, (UCSF), San Francisco, CA 94158, USA

**Keywords:** brain health, economics, transition, mental health, neurology

## Abstract

Optimal brain health is essential to smoothing major global skill-intensive economic transitions, such as the bioeconomy, green, care economy and digital transitions. Good brain health is vital to socio-economic sustainability, productivity and well-being. The care transition focuses on recognizing and investing in care services and care work as essential for economic growth and social well-being. The green transition involves shifting towards environmentally sustainable and fairer societies to combat climate change and environmental degradation. The digital transition aims to unlock digital growth potential and deploy innovative solutions for businesses and citizens, and to improve the accessibility and efficiency of services. The bioeconomy transition refers to the shift towards an economy based on products, services and processes derived from biological resources, such as plants and microorganisms. Brain capital, which encompasses brain health and brain skills, is a critical economic asset for the success of economies of the future. The brain economy transition from a brain-negative (brain-unhealthy) economy, which depletes brain capital, to a brain-positive (brain-healthy) economy, which arrests and reverses the loss of brain capital, will be foundational to these major transitions. Increased brain capital is vital to educational attainment, upskilling and reskilling. In this paper, we provide a detailed roadmap for the brain economy transition.

## Teaser

Nail-Beatty *et al*. report that optimal brain health is essential to smoothing major skill-intensive economic transitions, such as the bioeconomy, green, care economy and digital transitions. Good brain health is vital to socio-economic sustainability, productivity and well-being. They provide a detailed roadmap for the brain-positive economy transition.

## Graphical Abstract

**Graphical Abstract:**
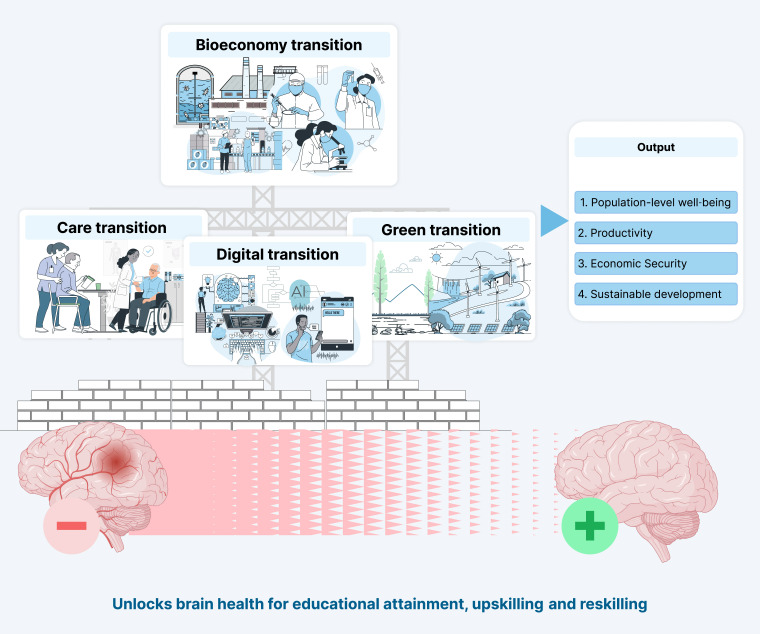

## Introduction

A 2015 paper from Hippe and Fouquet^1^ investigated how industrialized economies managed to achieve the transition from low to high levels of human capital and to identify lessons for green growth. They noted

The first phase of the human capital transition was the result of the interaction of supply and demand, triggered by technological change and boosted by the demands for (immaterial) services. The second phase of the human capital transition (i.e. mass education) resulted from enforced legislation and major public investment. The state’s aim to influence children’s beliefs appears to have been a key driver in public investment. Nevertheless, the roles governments played differed according to the developmental status and inherent socioeconomic and political characteristics of their countries. These features of the human capital transition are directly relevant to future transitions associated with green growth, and highlight the importance of understanding governments’ incentives and roles in transitions.

This paper extends this work by looking at the importance of social, emotional and cognitive brain resources in the future of work, and the current major brain health challenges societies are facing. We also extend beyond the green transition to look at other key transitions.

A global transition to a brain-positive economy that prioritizes brain capital will address the social, scientific and financial shifts necessary to minimize brain health disparities, ensuring equitable and productive participation in a global economy.^2,3^

### Brain capital for well-being, socio-economic sustainability and productivity

Our current brain-negative economy under-invests in brain capital, resulting in societies unable to capitalize on opportunities presented by current major economic transitions.^2,3^ Brain capital is a complex and productive stock composed of multidimensional factors that accumulate over the lifespan. The Global Brain Capital Dashboard identifies key drivers of brain health (i.e. neurological and mental health), including food and nutritional security, lack of prenatal healthcare and general health services, the natural and cultural environment, and education. When we do not prioritize healthy brains and individuals capable of acquiring new skills and fostering collective intelligence and resilience, our ability to innovate is hindered, slowing down and diminishing the quality of these transitions. We must transition to a brain-positive economy by prioritizing investment in these determinants and optimizing brain health by minimizing risk factors and enhancing protective factors.^3^ This transition is necessary to reverse the worldwide underinvestment in brain capital, and unlock the development of brain skills essential for major economic transitions and achieving the well-being and progress of the sustainable development goals.^4^

### The importance of brain skills in the modern economy

Addressing these issues now is critical, as today’s economy increasingly demands brain skills—cognitive, emotional and social skills—over motor and manual skills. Innovation as a deliverable of collective employee productivity is a growing expectation in the age of automation where lower level tasks are completed by artificial intelligence (AI) and cognitive skills are necessary for higher level thinking, cooperation and production.^3^ Within 5 years, according to the World Economic Forum’s 2023 Future of Jobs Report, employers will place a premium on cognitive and interpersonal skills to excel, adapt to changing environments and integrate new technologies into our lives. The development of these skills requires a healthy brain and greater social and workplace cohesion, which in turn requires a brain-positive economy.

### Major brain health challenges impacting brain capital

Major brain health challenges hampering vital brain skill development and deployment by individuals are not factored into economic transition strategy or planning. Challenges include neurological and mental disorders. One-in-three people will experience a neurological disorder, and nearly one in two will face a mental disorder at some point in their lives. Women and those in low- and middle-income countries are disproportionately affected. A recent study showed large-scale societal dynamics are related to brain structure and function. Lebedev *et al*.^5^ correlated large-scale UK biobank data over 14 years with the local stock market index (FTSE1000) and residents’ mood. They showed maximal stock market volatility was associated with volumetric measures of affective brain regions. Nearly $2 trillion US dollars are spent annually on neurological and mental disorders globally, increasing by a striking ∼5% each year. Yet, a 200 billion US dollar funding gap remains for mental disorders alone. This underinvestment drives a profound loss of brain capital.

### A structured approach to advancing the brain-positive economic transition

We previously described a four-step approach to enable stakeholders to swiftly mobilize around a shared national aspiration^2^: (i) develop a shared vision and mission for increasing brain capital, then explain and communicate with all stakeholders (including governments, civil society, private sector and philanthropy); (ii) identify and engage key drivers and champions; (iii) leverage and scale up existing brain initiatives and innovations and (iv) monitor and evaluate the progress and outcomes of the transition.

In this article, we aim to further refine our brain-positive economic transition model by demonstrating its intersection with other major economic transitions.

## The bioeconomy transition

This transition refers to the shift towards an economy based on products, services and processes derived from biological resources, such as plants and microorganisms. This transition is being actively advanced by the Biden Administration through initiatives like the Executive Order on Advancing Biotechnology and Biomanufacturing Innovation, which aims to promote sustainable, safe and secure biomanufacturing. This policy supports the development of a robust bioeconomy by setting bold goals, fostering innovation and ensuring responsible technology deployment. It is estimated that biotechnology applications in healthcare, including neuroscience, could have an economic impact of up to $1.3 trillion per year by 2030.

Key education and workforce considerations include expanding and diversifying the talent pool for biotechnology and biomanufacturing jobs, strengthening partnerships between employers, educational institutions and training providers, and developing innovative education and training approaches. These efforts are crucial for creating a skilled workforce capable of driving the bioeconomy forward.

This economic transition is essential for developing more advanced precision brain healthcare strategies. Enhanced diagnostic, treatment, early intervention and prevention solutions are critical for addressing brain health challenges such as depression, Alzheimer’s disease and bipolar disorder.^6^ Recent advancements in neuroscience research are fuelling the development of new psychiatric drugs and precision neuroscience techniques, which promise to revolutionize brain healthcare.

## The green transition

Public, private and civil society sectors are increasingly committing to environmental sustainability or ‘greener’ societies. A green transition includes a mixture of scientific, technological, business and policy innovations. The European Commission notes these innovations include scaling up alternative energy sources, improving the efficiency of resource consumption, sustainable manufacturing, scientific and technological advancements, sustainable agriculture, food systems and urban development, biodiversity conservation and ecosystem restoration, policy and regulatory support, and sustainable/systemic investing. Sub-transitions of the green transition include the clean energy transition and the nature-positive transition.

To foster this transition, both green skills and broader brain skills must be prioritized. According to the United Nations Industrial Development Organization, green skills include the knowledge, abilities, value and attitudes needed to live in, develop and support a sustainable and resource-efficient society.^7^ Achieving the broad system change required for a green transition will necessitate digital literacy and technological proficiency, as many green solutions rely heavily on digital technologies and data analysis, skills in policymaking, regulation and governance to create supportive frameworks for the green transition. Systems thinking skills to comprehend and manage the interconnected nature of environmental issues, entrepreneurial skills in driving eco-innovation and green business models, and behavioural insights for understanding human behaviour are critical in change management. We recently proposed a model of Green Brain Capital as an environmentally focused type of brain capital.^7^ We believe Green Brain Capital plays a pivotal role in this green transition by emphasizing the bidirectional relationship between brain health and the environment. It denotes both the process of expanding cognitive and environmental capabilities of the people and the level of their achieved well-being. This concept distinguishes between two sides of Green Brain Capital: the formation of capabilities, such as ecological intelligence, digital literacy, green skills and environmental determinants of brain health, and the use of these acquired capabilities, whether for work, leisure or contributing to a sustainable and resource-efficient society. The model addresses the complex challenge of assessing brain health’s environmental determinants and provides a framework for capturing the full spectrum of brain health across various domains. Additionally, it highlights the feedback loop between the green transition and brain health and finally aids in determining a country’s development and global positioning. Investing in green brain capital can prepare societies to address this transition.

### The clean energy transition

Energy production and consumption are responsible for a striking 75% of US emissions. Within the green transition, the clean energy transition focuses on reducing admissions and shifting from carbon-intensive to clean (carbon-free or carbon-limited) energy sources. The global goal as per the Paris Agreement is to realize net-zero CO_2_ emissions by 2050. To accomplish this goal, developing and mobilizing suitable human capital to meet workforce requirements is vital. This transition prioritizes skills, such as innovation, adaptability and complex problem-solving, all of which are crucial for workforce agility in adapting to new roles and implementing rapidly evolving technologies and infrastructure.

### The nature-positive transition

A nature-positive transition refers to a shift in economic activities and policies aimed at halting and reversing biodiversity loss while enhancing ecosystem and societal resilience. This approach goes beyond minimizing environmental damage to actively restoring and enriching natural habitats. It involves transforming business operations, value chains and financial systems to support biodiversity, store carbon, purify water and reduce pandemic risks.

The integration of natural capital accounting with economic decision-making processes, valuing ecosystem services and aligning financial flows with biodiversity conservation goals is essential. These nature-positive approaches emphasize the importance of circular economy principles and sustainable resource management. The necessary policy interventions, technological innovations, shifts in consumer behaviour and enhanced collaboration between governments, businesses and civil society to develop nature-based solutions and create markets that reward sustainable practices require significant investments in research, education and capacity building to cultivate the skills and knowledge needed for a nature-positive economy. For example, leveraging brain capital to drive innovation in areas such as biodiversity-friendly production methods or developing sophisticated monitoring/evaluation systems to track progress towards nature-positive outcomes, enabling adaptive management strategies.

### The effects of climate and the environment on brain health

Growing evidence suggests that factors related to climate change including exposure to ambient heat, environmental chemicals and air pollution can significantly impact brain function and lifelong health.^7^ Therefore, the green transition is key to unlocking brain health.

Rising temperatures and extreme weather events can exacerbate neurological conditions, such as stroke, multiple sclerosis, migraines and epilepsy.^8^ The effects of climate change-related factors on other chronic health conditions such as cardiovascular, respiratory and renal disorders, metabolic syndromes such as diabetes and obesity and mental health issues can also adversely affect brain function. Heat stress is associated with a range of neurological disruptions, including impaired neurodevelopment, slower cognitive and emotional processing, long-term losses in learning and memory, exacerbated symptoms of neurological and mental disorders, and increased permeability of the blood–brain barrier. Furthermore, early (including prenatal) exposure to extreme weather events increases the risks of anxiety, depression, attention deficit hyperactivity disorder (ADHD), post-traumatic stress disorder, diminished self-regulation and psychiatric disorders. Children and adolescents are particularly susceptible to environmental stressors due to their developing brains, immature physiological systems and increased blood–brain barrier permeability.^9^

We note two recent initiatives studying and developing solutions in the climate-brain interface. The Connecting Climate Minds programme recently published its ‘Global Research and Action Agenda for Climate Change and Mental Health’ and Columbia University’s Mailman School of Public Health recently launched a Neuro Climate Working Group, which has engaged diverse stakeholders.

### The feedback loop between the green transition and brain health

The green transition demonstrates how closely linked brain health is to other major transitions. An effective green and clean energy transition requires healthy brains and well-developed cognitive and interpersonal soft brain skills to drive innovation and implementation. However, factors related to climate change can negatively impact brain functioning, creating a barrier to progress. By prioritizing investment in brain capital alongside physical and natural capital, we can enhance cognitive abilities in the workforce, which in turn supports a smoother and more successful clean energy transition. This improved transition can also positively impact brain health by creating healthier environments, further advancing the cycle of mutual benefit.

### The Green Brain Capital

The Green Brain Capital, an environmentally focused type of brain capital, plays a pivotal role in this green transition by emphasizing the bidirectional relationship between brain health and the environment.^7^ It denotes both the process of expanding cognitive and environmental capabilities of the people and the level of their achieved well-being. This concept distinguishes between two sides of Green Brain Capital: the formation of capabilities, such as ecological intelligence, digital literacy, green skills and environmental determinants of brain health, and the use of these acquired capabilities, whether for work, leisure or contributing to a sustainable and resource-efficient society. The model addresses the complex challenge of assessing brain health’s environmental determinants and provides a framework for capturing the full spectrum of brain health across various domains. Additionally, it highlights the feedback loop between the green transition and brain health and finally aids in determining a country’s development and global positioning. Investing in green brain capital can prepare societies to address this transition.

## The care economy transition

The care economy encompasses the paid and unpaid activities, labor and relationships that sustain human activity. The economy is composed of community clinics, retirement homes, physiotherapy and childrearing. This transition includes a paradigm shift that recognizes care as a public good rather than as families’ or women’s responsibility. It requires incorporating an intersectional gender perspective into other major economic transition changes such as climate and just transition strategies in order to promote a more equitable and democratic distribution of care work, for example by incentivizing men and boys to take up care and domestic work and rewarding paid care work with decent jobs.

Social production encompasses unpaid contributions made by individuals and communities to society that underpin social cohesion, community resilience and collective well-being.^10^ Social production more broadly underpins the care economy transition by encompassing a wide range of unpaid activities, such as volunteering, childcare, care of the sick and elderly, informal mentoring, community participation and environmental restoration, that contribute to societal well-being. These activities, particularly unpaid childcare, form the backbone of the care economy and significantly influence the mental capital and mental health of future generations. Often, the largest contributors to social production are marginalized groups, such as the unemployed, older adults and those with disabilities, whose efforts are essential in maintaining community welfare. During crises, such as the COVID-19 pandemic, social production provides workforce surge capacity that supports care-related efforts and economic resilience, demonstrating its vital role in emergency response. Moreover, social production builds mental capital and collective well-being, which strengthen the care economy by enhancing the health and capabilities of both care providers and recipients. The care economy, particularly early childhood education and elderly care, is important in developing/maintaining cognitive abilities across the lifespan. It can enhance cognitive reserve, potentially reducing the risk of neurodegenerative diseases and improving overall brain health.^11^ Cognitive stimulation can help maintain brain health in older adults, reducing healthcare costs and extending productive engagement in society.^12^ Meanwhile, the care economy can also create brain-healthy environments that support optimal cognitive and emotional development in early childhood and adolescence, which have sustained effects on brain health outcomes across the life course.

Both social production and the care economy provide essential support to the formal economy by ensuring a brain-healthy, cohesive and more capable workforce and reducing the burden on formal healthcare and social services. By addressing mental health and social cohesion, these systems help maintain high levels of productivity and reduce economic costs related to healthcare, absenteeism and social unrest.^13^ As formal volunteering declines, some unpaid work may require a transition to paid care work, placing a greater burden on governments to fund these essential services. This, in turn, boosts economic productivity and national resilience, creating a more robust and prosperous formal economy.

## The digital transition

Digital technologies present enormous growth potential for nations. The digital transition aims to unlock digital growth potential and deploy innovative solutions for businesses and citizens and to improve the accessibility and efficiency of public services. The European Commission has a programme ‘A Europe fit for the digital age’ which aims to empower people, businesses and administrations with a new generation of technologies, where the digital transformation will benefit everyone.

The foundation of the digital transition lies in robust broadband internet infrastructure. High-speed internet access is essential for equitable participation in the digital economy, yet as of 2021 less than two-thirds of the global population had internet access. Heavy investments in infrastructure and human capital, heightened risks of cyberattacks and potential for system failures require robust safeguarding practices and cybersecurity measures to protect digital assets and ensure the resilience of digital economies. These interventions in themselves require brain capital.

The Center for European Policy Studies and Google recently collaborated to produce an Index of Readiness for Digital Lifelong Learning in Europe. The Index is composed of three ‘pillars’—composite indicators developed to capture the different dimensions and challenges of digital learning: (i) individual’s learning outcomes, (ii) availability of digital learning and (iii) institutions and policies for digital learning. Estonia was noted as the #1 country by this index. CEPS and Google also provided actional policy approaches for European nations.

Gen-AI is poised to significantly impact our brains in the coming decades, with both positive and negative effects. On the positive side, gen-AI can enhance cognitive functions by providing tools that aid in problem-solving, decision-making and learning. For instance, gen-AI-driven educational platforms can offer personalized learning experiences, helping individuals grasp complex concepts more effectively. However, there are potential negative effects as well. A recent article highlighted concerns that reliance on AI, particularly large language models, could diminish our cognitive abilities.^14^ It hypothesized that overdependence on gen-AI for tasks, such as writing and critical thinking, could lead to a decline in these soft brain skills, as our brains may become less engaged in these activities. Additionally, the ease of access to information through gen-AI could reduce our ability to retain and recall information, potentially impacting memory and learning processes. Balancing gen-AI use with active cognitive engagement will be crucial to mitigate these risks. Beyond gen-AI, it is important to consider the neuroplastic effects of prolonged digital engagement, understanding the long-term impacts of digital technologies on cognitive development across the lifespan and developing interventions to promote beneficial neuroadaptation.

To cope with this rapid incorporation of gen-AI technologies, society must prioritize expanding (rather than diminishing) our human capabilities and developing attributes in critical thinking, complex problem-solving, creativity, learning agility and adaptability, emotional intelligence, ethical judgement and integrity. The transition will also require technical skills, such as data literacy and analysis, design thinking and systems approaches, and change management. A systematic and forward-looking research and innovative strategy are crucial for a more productive and brain-positive economy, and will help ensure that society reaps the benefits of digitalization while confronting and bettering itself from its challenges.

## Effects of mental and neurological disorders and promotion of brain health on educational attainment, upskilling and reskilling

### Mental health challenges

Mental health challenges significantly impact educational attainment, upskilling and reskilling with profound economic consequences. Mental disorders such as anxiety, depression and bipolar disorders are associated with lower educational attainment as determined by high school and college graduation.^15^ These challenges can hinder cognitive functions, concentration and motivation, leading to lower academic performance. Critically, however, they impact not only immediate academic performance but also have long-term consequences for career prospects and lifetime earning potential.

In the context of upskilling and reskilling, mental health issues can be a substantial barrier, with impacts on memory, executive function and attention. Mental health problems can reduce an individual’s ability to engage in continuous learning and professional development. Stress, burnout and anxiety can diminish the capacity to acquire new skills, adapt to changing job requirements, pursue career advancement opportunities and live a meaningful and fulfilling life. Fostering mental health and well-being in a proactive and preventative way is thus crucial for fostering a supportive environment that promotes lifelong learning, career growth and biopsychosocial well-being.

### Neurological challenges

Neurological challenges significantly impact educational attainment, upskilling and reskilling. According to the RAND report neurodivergent individuals, such as those with autism, ADHD and dyslexia, often face barriers in traditional educational settings. These barriers can lead to lower academic performance and reduced opportunities for higher education, if not appropriately accommodated. While neurological differences can present challenges, they can also contribute to cognitive diversity, increasingly recognized as a valuable asset in innovation and problem-solving.

In the context of upskilling and reskilling, neurological challenges can also pose significant obstacles. Another RAND report highlights that cognitive impairments, such as dementia, can hinder the ability to learn new skills and adapt to changing job requirements. This is particularly concerning in fields that require continuous learning and adaptation. Addressing these challenges through inclusive educational practices and supportive workplace environments is crucial for enabling individuals with neurological conditions to achieve their full potential.

### The benefits of education on the brain

Education profoundly benefits the brain, enhancing cognitive functions and mental health. A recent study highlights that continuous learning through upskilling and reskilling can improve mental agility and adaptability.^16^ Educational activities stimulate neural pathways, promoting brain plasticity and cognitive resilience. Higher educational attainment is associated with increased grey matter volume in regions critical for executive function and memory, translating to improved cognitive performance, particularly in areas of complex reasoning and problem-solving.^17^ Individuals with higher levels of education have a significantly reduced risk of developing dementia, highlighting education’s role in developing a cognitive reserve.^18^ These effects appear to result from educational attainment contributing to differences in early adulthood that persist into old age, rather than attenuating the rate of cognitive decline in later life,^19^ further emphasizing the importance of early and continuous education.

It is well known that higher levels of education are associated with better mental health outcomes. Education provides individuals with a greater sense of control over their lives, thus reducing stress and anxiety. It plays a pivotal role in developing social cognition and emotional intelligence. Higher education levels are associated with enhanced theory of mind and empathy, key soft skills for social interaction and psychological well-being.^20^ Education also fosters critical thinking and problem-solving skills, which are essential for mental well-being. Additionally, educated individuals tend to have better socio-economic status, which further contributes to improved mental health by providing access to resources and opportunities that support a healthy lifestyle. Overall, education is a key factor in maintaining and enhancing brain health and soft brain skills.

### The social and economic determinants of brain health

A broad range of social and environmental factors influence brain health.^21^ Such factors include adverse early life stressors (e.g. abuse, maltreatment and neglect), substance misuse/abuse, exposure to family and community violence, unemployment, financial insecurity, poverty, poor education quality, homelessness, inequality, racism, social exclusion, natural disasters, climate change and other social and environmental factors that have unidirectional and bidirectional relationships with mental health and with each other in a complex causal web. In recognition of these important drivers of mental disorders, the World Health Organization’s comprehensive Mental Health Action Plan 2013–2030 calls for partnerships across health, education, employment, housing, social, private, judicial and other relevant sectors to deliver a comprehensive and coordinated response. The WHO’s Intersectoral Global Action Plan (IGAP) on epilepsy and other neurological disorders 2022–31 ‘implementation toolkit’ also provides a helpful roadmap for action. We recently published a report for the Bulletin of the WHO calling for a shift towards a well-being economy to better align commercial interests with collective well-being and social prosperity.^21^

## Well-being must be the product of the brain economy

Well-being is not merely a by-product but a crucial output of the brain economy transition and other significant economic shifts, including the green, digital, care and bioeconomy transitions. The brain economy, with its focus on cognitive health and skills, is inherently designed to foster overall mental and emotional well-being. Similarly, the clean energy and green transitions aim to create sustainable environments that support physical and mental health by reducing pollution and mitigating climate change impacts.

The digital transition, while presenting its own challenges, enhances access to information and services, promoting cognitive agility and social connectivity. The care economy focuses on providing essential health and social services, directly improving quality of life across the lifespan. Recent advances by the World Health Assembly, particularly through the Special Resolution on Economics of Health for all, emphasize the economic benefits of investing in health and the commitment to reshaping economies to deliver more holistic and equitable prosperity. Improved health outcomes lead to reduced reliance on social welfare and economic growth, underscoring the importance of well-being as a fundamental goal in these transitions.

By prioritizing well-being across all the transitions occurring in the 21st century, we can create a more resilient, innovative, sustainable, healthy and equitable society. Investment in brain capital not only fosters individual growth and potential but also drives broader economic and social progress, ensuring a healthier, more productive and sustainable future for all.

## Recommendations

The complex interplay of brain health, brain skills, the economy and the environment calls for tackling research, public, practice and financing challenges. Finding solutions to these challenges requires short-term (i.e. 0–5 years) and long-term (i.e. 5–15 years) efforts across disciplines, leveraging new technologies and interdisciplinary approaches to deepen our understanding and enhance our capacity for effective interventions.

### Research and innovation

Short-term actions (0–5 years)

- Fund studies examining the impact of built environments on brain health, particularly concerning neurodiversity and cognitive function, to develop evidence-based design guidelines
- Launch pilot projects applying brain-positive design principles in diverse settings—such as schools, workplaces and healthcare facilities—to demonstrate benefits and gather data for broader application
- Advanced biomonitoring technologies for neurological and mental health disorders in the context of individuals’ exosome and usage of AI technologies
- Conduct large-scale longitudinal studies to understand the effects of exposome, social and economic determinants on brain health
- Conduct interdisciplinary studies to connect neuroscience with economics and environmental studies
  - Understand the impact of brain health on economic productivity
  - Understand the interplay of brain health with individuals’ exposomes (connected with the above)
- Develop and support pilot programmes that test innovative approaches to enhancing brain health and soft skills development in different populations
- Improve data collection and analysis strategies on brain capital and Mental Wealth metrics to best identify trends, disparities and effective interventions

Long-term actions (5–15 years)

- Invest in long-term research initiatives exploring the relationship between built environments and brain health, focusing on innovative materials, design approaches and technologies
- Facilitate the large-scale application of brain-positive design principles through public–private partnerships and collaborative initiatives to translate research findings into practical solutions
- Translate research findings into predictive models for brain health outcomes
- Develop personalized intervention strategies based on brain health metrics and exposome data
- Invest in longitudinal studies to track the effects of early brain health interventions on life outcomes and economic participation
- Foster international collaborations to share data, research findings and best practices in brain health and economic development

### Research and innovation

Short-term actions (0–5 years)

- Fund studies examining the impact of built environments on brain health, particularly concerning neurodiversity and cognitive function, to develop evidence-based design guidelines
- Launch pilot projects applying brain-positive design principles in diverse settings—such as schools, workplaces and healthcare facilities—to demonstrate benefits and gather data for broader application
- Advanced biomonitoring technologies for neurological and mental health disorders in the context of individuals’ exosome and usage of AI technologies
- Conduct large-scale longitudinal studies to understand the effects of exposome, social and economic determinants on brain health
- Conduct interdisciplinary studies to connect neuroscience with economics and environmental studies
  - Understand the impact of brain health on economic productivity
  - Understand the interplay of brain health with individuals’ exposomes (connected with the above)
- Develop and support pilot programmes that test innovative approaches to enhancing brain health and soft skills development in different populations
- Improve data collection and analysis strategies on brain capital and Mental Wealth metrics to best identify trends, disparities and effective interventions

Long-term actions (5–15 years)

- Invest in long-term research initiatives exploring the relationship between built environments and brain health, focusing on innovative materials, design approaches and technologies
- Facilitate the large-scale application of brain-positive design principles through public–private partnerships and collaborative initiatives to translate research findings into practical solutions
- Translate research findings into predictive models for brain health outcomes
- Develop personalized intervention strategies based on brain health metrics and exposome data
- Invest in longitudinal studies to track the effects of early brain health interventions on life outcomes and economic participation
- Foster international collaborations to share data, research findings and best practices in brain health and economic development

### Public policy

Short-term actions (0–5 years)

- Discuss and arrive at a consensus on brain economy in intergovernmental fora, such as the Yaoundé Declaration on the Brain Economy^22^
- Support the implementation of the WHO IGAP on epilepsy and other neurological disorders 2022–31: implementation toolkit
- Identify exposome and brain health research components that enable actionable public health strategies and regulatory policies
- Develop educational programmes to increase public awareness of the exposome, gen-AI technologies and brain health and its impact on economic productivity
- Train transdisciplinary-based neuroscientists and healthcare providers
- Develop national brain health plans that incorporate comprehensive mental health coverage, leveraging models such as the Swiss and Finnish brain health plans
- Incorporate brain health into Environmental, Social and Governance (ESG) frameworks and provide tax incentives and grants for businesses and organizations that implement brain health programmes and policies
- Explore linkages between the brain economy transition and other key economic challenges, such as global trade structures, welfare systems, banking systems, illicit financial flows and global taxation

Long-term actions (5–15 years)

- Invest in fostering brain capital the status quo for businesses by integrating brain health as a central component to public health policies, ensuring long-term funding and support for brain health initiatives
- Develop and invest in toolkits describing specific actions and resources for countries to improve services for people with neurological disorders, such as the IGAP
- Enact global policies and agreements to reduce harmful environmental exposures, lower CO_2_ emissions and regulate AI technologies
- Develop a comprehensive policy framework with a life course approach; addressing brain health from pre-conception through old age, integrating education, healthcare and workplace regulations
- Monitor and evaluate the effectiveness of these new policies

### K-12 education (encompassing primary, middle and high school)

Short-term actions (0–5 years)

- Ensure universal education noting illiteracy rates in Africa, South East Asia and the Middle East are still 20% or more
- Create training programmes for teachers on how to support student’s brain health and interpersonal skills development through classroom practices and early intervention strategies for mental health and neurological disorders
- Integrate brain-positive design concepts into school environments to enhance learning outcomes and student well-being, including designing classrooms that support cognitive development and reduce stress
- Develop regulations on the use of certain technologies in the classroom, including determination on how and when gen-AI can be used
- Educate the global youth on green skills and brain health
- Expand access to mental health services within schools to support students through to graduation

Long-term actions (5–15 years)

- Develop and adopt holistic education models that prioritize problem-solving, creativity and emotional intelligence alongside traditional academic skills
- Advanced remote and personalized learning tools into the Global South, and measure and optimize effectiveness

### Workplace

Short-term actions (0–5 years)

- Develop workplace training programmes and encourage continuous learning to support cognitive skill development and adaptation to new job requirements
- Implement programmes or frameworks to improve brain health, including promoting a healthy work-life balance, providing mental health resources (like mental health days) and monitoring workload and stress levels
- Improve workplace social and emotional ergonomics
- Support pioneering programmes such as the Business Collaborative for Brain Health

Long-term actions (5–15 years)

- Incorporate brain health into ESG frameworks and provide tax incentives and grants for businesses and organizations that implement brain health programmes and policies
  - Create tax incentives and grants for businesses that prioritize the green transition as well
  - Promote a culture of holistic health: develop integrated health and productivity programmes that link employee well-being with organizational performance
- Create pathways for ongoing professional development that focus on cognitive skills, emotional intelligence and adaptability to support lifelong learning and career advancement

### Technology

Short-term actions (0–5 years)

- Employ AI for data analysis and technology development for brain health
  - Leverage AI to develop tools that can identify early signs of mental health issues and provide personalized interventions and support
- Promote the development and use of mental health and cognitive training apps to support individuals in managing stress, anxiety and improving cognitive functions (especially in conditions that affect social interactions and emotional regulation such as autism and ADHD)
- Fund and implement digital literacy programmes that teach individuals how to effectively use technology and promote awareness for the role of technology in our lives
- Guarantee an adequate regulatory framework tailored to different nations and regions

Long-term actions (5–15 years)

- Develop and implement smart city technologies that reduce environmental exposures and reduce emissions
  - Develop smart environments (homes, schools, workplaces) equipped with technology that supports cognitive and emotional well-being through personalized and adaptive systems (like a home system that minimizes blue light after a certain time)
- Invest in the development of non-invasive, wearable devices that monitor brain health indications and provide real-time feedback to users to enable healthy living

### Finance

Short-term actions (0–5 years)

- Create a taxonomy of funding instruments:
  - Develop a comprehensive taxonomy categorizing tools and mechanisms available for financing projects, initiatives or organizations. This taxonomy will cover various funding sources across the capital stack, including equity, debt, grants, revenue-based financing, donations and philanthropy, hybrid instruments, specialized funding instruments and impact bonds
- Create data-based case for brain capital interventions:
  - Collect and analyse data to build a compelling case for interventions that preserve brain capital. This involves demonstrating how optimizing treatments and care can save human and economic resources
- Support pilot projects:
  - Launch and evaluate pilot projects to showcase the value of anticipatory and preventive actions. Initial pilots could include:
    - Sustaining and Scaling Mental Health Initiatives: expand impactful approaches like The Friendship Bench
    - Dementia Care Services: increase the availability of services and integrate new treatments, such as monoclonal antibodies, into existing systems
    - MS Data Registry: establish a comprehensive registry for multiple sclerosis, including deep phenotyping and longitudinal follow-up, to enable large-data studies and identify early markers
- Convene stakeholders:
  - Organize a convening to advance the Brain Economy Funding Plan
- Explore the role of gender lens investing in the brain economy investment framework

Long-term actions (5–15 years)

- Sustain implementation of funding taxonomy:
  - Utilize the developed taxonomy to streamline and optimize funding for brain health projects. Encourage adoption by policymakers, investors and funding organizations to ensure diverse and sustainable financial support
- Create and support comprehensive brain capital strategies:
  - Implement large-scale interventions based on the data-driven insights gathered in the short term. Optimize treatments and care practices across healthcare systems to maximize human and economic benefits
- Support expanded and enhanced pilot projects:
  - Scale successful pilot projects to broader regions and populations. Examples include:
    - Global Dementia Care: Further integration and expansion of advanced dementia treatments
    - MS Research and Treatment: Utilize the MS Data Registry for international research collaborations and improved patient outcomes
    - Risk Factor Research: Conduct global studies to identify modifiable risk factors for brain disorders, aiming to mitigate health and economic losses
- Create and support a neurotechnology medicine platform:
  - Establish and develop the Neurotechnology Medicine Platform to foster advancements in the pre-competitive space. This platform will drive innovation and collaboration in neurotechnology, ultimately leading to breakthroughs in brain health
- Foster and sustain global collaboration and policy development:
- Foster international collaboration to develop and implement policies supporting brain health initiatives. Use the findings and successes from pilot projects and data analysis to inform global health policies and investment strategies

## Conclusion

Our economic system must integrate nature, internalize externalities, value care and adapt to technological change. It must aim for well-being, socio-economic sustainability and productivity. The brain-positive economic transition is not just another initiative; rather, it is the transition that enables all other transitions. Adopting these goals will create an economic system that centres around the people, is sensitive to climate and the natural environment and ensures that technology serves the interests of society. By prioritizing brain health and brain skills, we can build a more resilient, innovative, healthy, sustainable and equitable economy and society that supports the myriad transitions our world is presently undergoing and will undergo in the future. Indeed, we believe this holistic, brain health–centred approach is essential for achieving long-term well-being and prosperity for all individuals and societies worldwide.

## Data Availability

Data sharing is not applicable to this article as no new data were created or analysed in this study.
